# Cell fixation and preservation for droplet-based single-cell transcriptomics

**DOI:** 10.1186/s12915-017-0383-5

**Published:** 2017-05-19

**Authors:** Jonathan Alles, Nikos Karaiskos, Samantha D. Praktiknjo, Stefanie Grosswendt, Philipp Wahle, Pierre-Louis Ruffault, Salah Ayoub, Luisa Schreyer, Anastasiya Boltengagen, Carmen Birchmeier, Robert Zinzen, Christine Kocks, Nikolaus Rajewsky

**Affiliations:** 1 Systems Biology of Gene Regulatory Elements, Berlin Institute for Medical Systems Biology (BIMSB), Max Delbrück Center for Molecular Medicine in the Helmholtz Association (MDC), 13125 Berlin, Germany; 2 Systems Biology of Neural Tissue Differentiation, Berlin Institute for Medical Systems Biology (BIMSB), Max Delbrück Center for Molecular Medicine in the Helmholtz Association (MDC), 13125 Berlin, Germany; 3 Developmental Biology/Signal Transduction, Max Delbrück Center for Molecular Medicine in the Helmholtz Association (MDC), 13125 Berlin, Germany

**Keywords:** Droplet-based single-cell transcriptomics, Drop-seq, Gene expression profiling, Fixation, Alcohol-based fixation, Methanol, Fluorescent activated cell sorting (FACS), Primary cells, Tissue

## Abstract

**Background:**

Recent developments in droplet-based microfluidics allow the transcriptional profiling of thousands of individual cells in a quantitative, highly parallel and cost-effective way. A critical, often limiting step is the preparation of cells in an unperturbed state, not altered by stress or ageing. Other challenges are rare cells that need to be collected over several days or samples prepared at different times or locations.

**Methods:**

Here, we used chemical fixation to address these problems. Methanol fixation allowed us to stabilise and preserve dissociated cells for weeks without compromising single-cell RNA sequencing data.

**Results:**

By using mixtures of fixed, cultured human and mouse cells, we first showed that individual transcriptomes could be confidently assigned to one of the two species. Single-cell gene expression from live and fixed samples correlated well with bulk mRNA-seq data. We then applied methanol fixation to transcriptionally profile primary cells from dissociated, complex tissues. Low RNA content cells from *Drosophila* embryos, as well as mouse hindbrain and cerebellum cells prepared by fluorescence-activated cell sorting, were successfully analysed after fixation, storage and single-cell droplet RNA-seq. We were able to identify diverse cell populations, including neuronal subtypes. As an additional resource, we provide 'dropbead', an R package for exploratory data analysis, visualization and filtering of Drop-seq data.

**Conclusions:**

We expect that the availability of a simple cell fixation method will open up many new opportunities in diverse biological contexts to analyse transcriptional dynamics at single-cell resolution.

**Electronic supplementary material:**

The online version of this article (doi:10.1186/s12915-017-0383-5) contains supplementary material, which is available to authorized users.

## Background

A tissue is composed of many specialized cell types, each of which can have various biological states. Rather than studying global gene expression of a tissue as a whole, it has been recognized that transcriptional profiling at a single-cell resolution [1–4] provides a much more complete and accurate description of its biological function [5, 6]. Recent advances in droplet-based microfluidic technologies have made it possible to capture, index and sequence the transcriptional profiles of thousands of individual cells in a highly parallel, ultrafast and affordable manner [7, 8].

In the ‘Drop-seq’ method described by Macosko et al. [7], cells are separately encapsulated in nanoliter-sized droplets together with a single bead in a microfluidic device. One bead delivers barcoded primers, each harbouring a polymerase chain reaction (PCR) handle, a cell barcode and a multitude of different unique molecular identifiers (UMIs), followed by a polyT sequence. The beads are suspended in a lysis buffer, resulting in the cell being lysed upon droplet formation. Cellular messenger RNAs (mRNAs) are released and can hybridize to the polyT sequences of the barcoded bead primers. After collection, the droplets are broken and the mRNA is reverse transcribed into complementary DNA (cDNA), PCR-amplified and sequenced in bulk. Computational analysis allows us to distinctly assign which mRNAs originate from the same cell by means of the cell barcode. The UMIs are used to identify and remove PCR duplicates and to digitally count distinct mRNA molecules.

Despite the rapid rise in high-throughput single-cell RNA-sequencing (RNA-seq) methods, including commercialized versions of automated platforms such as the Fluidigm C1, 10XGenomics or 1CellBiO systems, comparatively little attention has been given to the limitations that need to be overcome in the preparation and handling of cellular input material [9]. A major challenge in obtaining meaningful information is the use of a high-quality single-cell suspension which appropriately reflects the transcriptional state of each cell within its natural or experimentally intended environment. The steps between cell harvesting from culture or after tissue dissociation, isolation of single cells and mRNA capture are particularly critical as they are prone to introduce transcriptome changes and degradation of RNA. Requirements such as the need to pool cells from several tissues or culture conditions, possibly combined with time course experiments, represent an additional restriction.

In principle, many of these problems could be addressed with the help of chemical fixation. Unlike aldehydes, methanol and ethanol are coagulating fixatives that do not chemically modify nucleic acids [10, 11]. Alcohols act by dehydration: in higher than 65% alcohol and in the presence of salts, nucleic acids occur in a collapsed state that can be reverted to its original form by a simple rehydration. We have previously shown that fixation with 80% methanol is compatible with next-generation sequencing and library preparation for both mRNAs and small RNAs [12]. Fixation was critically required for successful genome-wide gene expression profiling of sorted, one- to four-cell stage *Caenorhabditis elegans* embryos, a complex tissue undergoing rapid and dynamic transcriptional changes [12].

Here, we adapted the methanol-based fixation protocol from Stoeckius et al. [12] to preserve cells for subsequent profiling of single-cell transcriptomes by Drop-seq. We first analysed both live and fixed mixtures of cultured human (HEK) and mouse (3T3) cells to demonstrate that methanol fixation does not change the numbers of genes and transcripts (defined as the number of UMIs) detected per cell or interfere with unambiguous assignment of reads to one or the other species. We then applied methanol fixation to a larger scale analysis of ~9000 primary cells from dissociated *Drosophila* embryos or sorted mouse hindbrain cells. We demonstrate that Drop-seq profiling of single-cell transcriptomes with methanol-fixed cells performs well with both cultured and primary cells.

Additionally, we provide a computational resource to facilitate the exploration of droplet-based single-cell sequencing data. 'dropbead' can be readily used to visualize basic statistics and quantitative parameters, compare different samples and filter samples prior to subsequent analysis.

## Methods

### Preparation and fixation of cell lines for Drop-seq

Human Flp-In T-Rex 293 HEK cells were a gift from M. Landthaler (Max Delbrück Center for Molecular Medicine in the Helmholtz Association (MDC), Berlin) originally obtained from Invitrogen (catalog no. R78007); murine NIH/3T3 cells were from DSMZ (ACC 59, DSMZ, Braunschweig, Germany). Cells were grown in Dulbecco’s modified Eagle’s medium (DMEM, 61965-026, Invitrogen, Waltham, MA, USA) without antibiotics containing 10% fetal bovine serum and confirmed to be mycoplasma-free (LookOut Mycoplasma PCR detection kit, Sigma-Aldrich, St. Louis, MO, USA). Cells were grown to 30–60% confluence, dissociated for 5 min at 37 °C with 0.05% bovine trypsin-EDTA (Invitrogen 25300062), quenched with growth medium and further processed as described previously (Macosko et al. [7, 13]). Briefly, between ~1 and 10 × 10^6^ cells were handled always in the cold and kept on ice, pelleted at 300 × g for 5 min at 4 °C, washed with 1× phosphate-buffered saline (PBS) + 0.01% bovine serum albumin fraction V (BSA) (100 μg/ml; 01400, Biomol, Hamburg, Germany), resuspended in PBS, filtered through a 40- or 35-μm cell strainer and counted. For Drop-seq, a [1 + 1] mixture of [HEK + 3T3 cells] was prepared at a combined input concentration of 100 cells/μl in 1× PBS + 0.01% BSA (corresponding to a final concentration of 50 cells/μl after mixing with lysis buffer in the co-flow device).

Methanol fixation was adapted from Stoeckius et al. [12]. Cells were trypsinized, and between 1 and 4 × 10^6^ cells were processed as described above for Drop-seq [7]. Cells were handled in regular (not ’low-binding’) microcentrifuge tubes to minimize cell loss and kept cold at all times. After straining and counting, cells were pelleted at 300 × g for 5 min at 4 °C, the supernatant was removed manually and the cell pellet resuspended in 2 volumes (200 μl) of ice-cold PBS. To avoid cell clumping, 8 volumes (800 μl) of methanol (grade p.a.; pre-chilled to –20 °C) were added dropwise, while gently mixing or vortexing the cell suspension (final concentration: 80% methanol in PBS). The methanol-fixed cells were kept on ice for a minimum of 15 min and then stored at –80 °C for up to several months, as indicated. For rehydration, cells were either kept on ice after fixation (Fixed) or moved from –80 °C to 4 °C (Fixed 1 or 3 weeks) and kept in the cold throughout the procedure. Cells were pelleted at 1000 to 3000 × g, resuspended in PBS + 0.01% BSA, centrifuged again, resuspended in PBS + 0.01% BSA, passed through a 40- or 35-μm cell strainer, counted and diluted for Drop-seq in PBS + 0.01% BSA as described above. For control of RNA quality after fixation, cells were resuspended in PBS, kept on ice for 5–10 min and repelleted; RNA was then extracted with TRIZOL.

### Preparation of *Drosophila* cells for Drop-seq

The *D. melanogaster* strain used was *y* ^*1*^ *w* ^*1118*^ *; P{st.2::Gal4}; P{vnd::dsRED}* [14]. Eggs were collected on apple juice-agar plates for 2 h and aged for ~6 h at 25 °C. Embryos were dechorionated for 1 min in ~4% NaOCl (diluted commercial bleach) and extensively washed with deionized water. Excess liquid was removed, and embryos were transferred to 1 ml ice-cold dissociation buffer (cell culture grade PBS, 0.01% molecular biology grade BSA) 6 h after embryo collection. Approximately 500–1000 embryos were collected prior to dissociation; a small subsample was stored in methanol for later staging by microscopy. Embryos were dissociated in a Dounce homogenizer (Wheaton 357544) with gentle, short strokes of the loose pestle on ice until all embryos were disrupted. The suspension was transferred into a 1.5-ml microcentrifuge tube, and the cells were pelleted for 3 min at 1000 × g at 4 °C. The supernatant was exchanged for 1 ml fresh dissociation buffer. Cells were further dissociated using 20 gentle passes through a 22G x 2” needle mounted on a 5-ml syringe. The cell suspension was then gently passed through a 20-μm cell strainer (NY2002500, Merck, Darmstadt, Germany) into a fresh 1.5-ml microcentrifuge tube, and residual cells were washed from the strainer using a small amount of dissociation buffer. Cells were pelleted for 3 min at 1000 × g at 4 °C and resuspended in 100 μl fresh dissociation buffer and counted. Samples were fixed by adding 4 volumes of ice-cold 100% methanol (final concentration of 80% methanol in PBS) and thoroughly mixed with a micropipette. Cells were stored at –20 °C until use (for up to 2 weeks).

For Drop-seq runs, cells were moved to 4 °C and kept in the cold throughout the procedure. Cells were pelleted at 3000 × g for 5 min, rehydrated in PBS + 0.01% BSA in the presence of RNAse inhibitor (RiboLock 1U/μl), pelleted and resuspended again in the presence of RNAse inhibitor, passed through a 35-μm cell strainer, counted and finally diluted for Drop-seq into PBS + 0.01% BSA (final concentration of 50 cells/μl).

### Preparation of mouse hindbrain cells for Drop-seq

Brains of newborn mouse pups (C57BL/6; postnatal day 5) were dissected in ice-cold buffer (120 mM NaCl, 8 mM KCl, 1.26 mM CaCl_2_, 1.5 mM MgCl_2_, 21 mM NaHCO_3_, 0.58 mM Na_2_HPO_4_ and 30 mM glucose, pH 7.4) that was saturated with 95% O_2_ and 5% CO_2_. Transsections at the level of the pons and C2 motor roots were performed using a razor blade to isolate the rhombencephalon. Hindbrain and cerebellar tissues were dissociated using the Papain Dissociation System (Worthington Biochemical, Lakewood, NJ, USA) according to the manufacturer's instructions. To facilitate dissociation and prevent aggregation, DNAse I (5U/ml, Roche, Basel, Switzerland) was added to the protease solution. After inactivation, cells were resuspended in Mg^2+^- and Ca^2+^-free Hank's Balanced Salt Solution. Live (propidium iodide-negative) cells were sorted directly into ice-cold methanol (final concentration 80% methanol), and the fixed cells were stored for more than 4 weeks at –80 °C. The sort was carried out under low pressure flow settings (23 psi; 100-μm nozzle), previously optimized to maximize recovery of viable cells for subcultures. For Drop-seq, aliquots with 10^6^ (replicate 1) or 3 × 10^5^ (replicate 2) sorted, methanol-fixed cells from three or two hindbrains, respectively, were pelleted and processed as described above. RNAse inhibitor (RiboLock 1 U/μl) was added during the rehydration, wash and cell straining steps. Cell recovery was 19% and 12% from the two cell preparations, respectively. Cells were diluted 1:3 into PBS-BSA 0.01% and then used for Drop-seq.

### Drop-seq procedure, single-cell library generation and sequencing

Monodisperse droplets of about 1 nl in size were generated using microfluidic polydimethylsiloxane (PDMS) co-flow devices (Drop-seq chips, FlowJEM, Toronto, Canada; rendered hydrophobic by pre-treatment with Aquapel). Barcoded microparticles (Barcoded Beads SeqB, ChemGenes Corp., Wilmington, MA, USA) were prepared and, using a self-built Drop-seq setup, flowed in closely following the previously described instrument setup and procedures by Macosko et al. [7, 13]. For most microfluidic co-flow devices, emulsions were checked by microscopic inspection; typically less than 5% of bead-occupied droplets contained more than a single barcoded bead. Droplets were collected in one 50-ml Falcon tube for a run time of 12.5 min. Droplets were broken promptly after collection, and barcoded beads with captured transcriptomes were reverse transcribed without delay, then exonuclease-treated and further processed as described [7]. The first-strand cDNA was amplified (after assuming loss of about 50% of input beads) by equally distributing beads from one run to 24 or 48 PCR reactions (between 10 and 30 anticipated STAMPS per tube); 50 μl per PCR reaction; 4 + 9 cycles (except for mouse hindbrain replicate 2: 4 + 12 cycles). Then 20- or 10-μl fractions of each PCR reaction were pooled and double-purified with a 0.6× volume of Agencourt AMPure XP beads (catalog no. A63881, Beckman Coulter, Pasadena, CA, USA) and eluted in 12 μl H_2_O. We evaluated and quantified 1 μl of the amplified cDNA libraries on a BioAnalyzer High Sensitivity Chip (Agilent, Santa Clara, CA, USA). Then 600 pg of each cDNA library was fragmented and amplified (12 cycles) for sequencing with the Nextera XT v2 DNA Library Preparation kit (Illumina, San Diego, CA, USA) using custom primers that amplified only the 3' ends [7]. Libraries were purified with a 0.6× volume of AMPure XP beads followed by a 0.6–1× volume of AMPure beads to completely remove primer dimers and achieve an average length of ~500–700 bp, quantified and sequenced (paired end) on Illumina NextSeq 500 sequencers (library concentration: 1.8 pM; 1% PhiX spike-in for run quality control; NextSeq 500/550 High Output v2 kit (75 cycles); read 1: 20 bp (bases 1–12 cell barcode, bases 13–20 UMI; custom primer 1 Read1CustSeqB), index read: 8 bp, read 2 (paired end): 64 bp).

The unique identifiers are as follows: Live: GSM2359902; Fixed: GSM2359903; Fixed 1 week: GSM2359904; Fixed 3 weeks: GSM2359905; *Drosophila*: mel_rep1: GSM2518777; mel_rep2: GSM2518778; mel_rep3: GSM2518779; mel_rep4: GSM2518780; mel_rep5: GSM2518781; mel_rep6: GSM2518782; mel_rep7: GSM2518783; Mouse: mm_rep1: GSM2518784; mm_rep2: GSM2518785.

### Single-cell RNA-seq: data processing, alignment and gene quantification

We chose read 1 to be 20 bp long to avoid reading into the poly(A) tail, leaving 64 bp for read 2. The sequencing quality was assessed by FastQC (v.0.11.2). The base qualities of read 1 were particularly inspected, since they contain the cell and molecular barcodes and their accuracy is critical for the subsequent analysis. The last base of read 1 consistently showed an increase in T content, possibly indicating errors during bead synthesis. We observed a similar trend when re-analyzing the original data from Macosko et al. [7]. Part of these errors were handled and corrected as described later. For read 2, we used the Drop-seq tools v.1.12 [7] to tag the sequences with their corresponding cell and molecular barcodes, to trim poly(A) stretches and potential SMART adapter contaminants and to filter out barcodes with low-quality bases.

For mixed species experiments with human and mouse cells, the reads were then aligned to a combined FASTA file of the hg38 and mm10 reference genomes, using STAR [15] v.2.4.0j with default parameters. Typically, around 65% of the reads were found to uniquely map to either of the species genomes. The *Drosophila melanogaster* sequences were mapped to the BDGP6 reference genome; typically around 85% of the reads mapped uniquely. For the mouse hindbrain samples, 75% of all sequence reads mapped uniquely. Non-uniquely mapped reads were discarded.

The Drop-seq toolkit [7] was further exploited to add gene annotation tags to the aligned reads (the annotation used was Ensembl release 84) and to identify and correct some of the bead synthesis errors described above. The number of cells (cell barcodes associated with single-cell transcriptomes) was determined by extracting the number of reads per cell, then plotting the cumulative distribution of reads against the cell barcodes ordered by descending number of reads and selecting the inflection point (‘knee’) of the distribution. It was similar to the number of single-cell transcriptomes expected during the Drop-seq run (see Additional file 1: Figure S1 for details). Finally, the DigitalExpression tool [7] was used to obtain the digital gene expression (DGE) matrix for each sample.

### Exploratory analysis, visualization and filtering of Drop-seq data

We developed a freely available R software package ('dropbead'; including a tutorial), which offers an easy and systematic framework for exploratory data analysis and visualization. ’dropbead’ provides a function for computationally determining the inflection point and hence the number of cells in a sample. The starting point for subsequent analysis is the sample's DGE. ’dropbead’ provides functions for creating species separation plots and violin plots of genes and transcripts per cell. ’dropbead’ can be used to easily filter and remove genes with low counts and cells with few UMIs, or to keep the best cells according to a certain criterion. ’dropbead’ was used to generate Figs. 2a, b, c, 3a and 4a as well as Additional files 1, 2, 3 and 6: Figure S1a, b, f, Figure S2a, b, c, Figure S3a, b, Figure S5a, b.

We discarded cells from subsequent analysis which had fewer than 3500 UMIs (HEK and 3T3 cells), 1000 UMIs (*Drosophila* samples) or 300 UMIs (mouse samples). In the human-mouse mixed species experiments, a threshold of 90% (90 out of 100 UMIs for one species) was selected to confidently declare a cell as being either of the species and not a human/mouse doublet. In order to assess whether fixation generates ’low-quality cells’ [16], we determined the proportion of non-mitochondrial reads: for every cell we computed the sum of UMIs corresponding to RNA encoded by the mitochondrial genome and then subtracted this number from the sum of all UMIs in that cell. We divided this number by the total number of UMIs in the cell to obtain the non-mitochondrial content as a percentage for every sample.

### Single-cell RNA-seq: normalization and correlations of gene expression levels

The raw counts in the DGE matrix were normalized to average transcripts per million (ATPM) as follows: the UMI counts for every gene in a given cell were divided by the sum of all UMIs in that cell. These counts were then multiplied by the sum of all UMIs of the cell with the highest number of UMIs in that library. Correlations of gene expression levels between single-cell samples were computed by first subsetting the DGE matrices of the two samples to the intersection of the genes captured in both libraries and then computing the sum of gene counts across all cells in each library. Plotting of correlations is shown in log space. For the correlation of Drop-seq data against mRNA-seq, we converted gene counts into reads per kilobase per million (RPKMs) and used the mean value of all isoform lengths for a given gene. For all correlations, the intersection (common set) of genes was high, around 17,000 genes for human and mouse samples (cell lines and primary cells) and 10,000 genes for *D. melanogaster*.

### Single-cell RNA-seq: clustering and identification of cell populations and marker genes

For the *Drosophila* embryos and mouse hindbrain samples, after filtering our samples with ‘dropbead’ we used Seurat [17] for cluster analysis. We first identified a set of highly variable genes, which we used to perform principal component (PC) analysis. Judged by their statistical significance and the robustness of the results, the first few (about 20–50) PCs were subsequently used as inputs for clustering and subsequent *t*-distributed stochastic neighbour embedding (tSNE) representation. The clustering was performed with the function 'FindClusters' of Seurat using default parameters and 50 PCs (*Drosophila*) or 21 PCs (mouse) as input. The same number of PCs was used as input for the tSNE representation. We used Seurat’s function 'FindAllMarkers' to identify the marker genes for each of the clusters in the tSNE representation.

The initial clusterings for both the *Drosophila* embryos and the mouse hindbrain samples contained cell clusters which were difficult to characterize (three and one cluster, respectively). After closer inspection, we flagged two cell clusters in the *Drosophila* data as being nuclei, as cells were lacking substantial expression of mitochondrially encoded genes compared to the rest of the cells. We classified these cells as nuclei (probably generated by mechanic disruption in the cell isolation procedure) and excluded them from further analysis (2975 cells).

Furthermore, extrapolating from our mixed species experiments with human and mouse cell lines, we anticipated around 10–15% of same-species doublets for the *Drosophila* embryos and mouse hindbrain data sets. For *Drosophila*, we flagged a cluster of cells whose marker genes, as identified by Seurat, lacked specificity. For mouse, we flagged a similar cluster of cells which additionally contained higher portions of ribosomal protein coding mRNAs than the rest of the cells. We reasoned that both sets might be cell doublets and excluded them in order to perform the final cluster analysis shown in Figs. 3b and 4b (excluded cells: *Drosophila*, 2186 cells; mouse, 1127 cells).

### Bulk mRNA-seq libraries

Live, cultured cells (Flp-In T-Rex 293 HEK cells, NIH/3T3 cells), intact, live *Drosophila melanogaster* embryos and sorted, methanol-fixed cells from dissected newborn mouse hindbrain and cerebellum were used for total RNA extraction with TRIZOL. Strand-specific cDNA libraries were generated according to the Illumina TruSeq protocol (TruSeq Stranded mRNA LT Sample Prep Kit, Illumina) using between 24 to 260 ng of total RNA input. The 1.8-pM libraries were sequenced on an Illumina NextSeq 500 System, using the High Output v2 Kit (150 cycles), single read: 150 bp, index read: 6 bp.

The unique identifiers are as follows: bulk_hek: GSM2518786; bulk_3t3: GSM2518787; bulk_mel1: GSM2518788; bulk_mel2: GSM2518789; bulk_mm: GSM2518790.

## Results

### Methanol fixation preserves single-cell transcriptomes for droplet-based sequencing

#### Drop-seq with methanol-fixed cells allows correct species assignments in species-mixing experiments

In order to assess whether methanol fixation is compatible with Drop-seq, we adapted our previously developed methanol fixation protocol [12] to adherent, mammalian cell lines (see Methods for details). Methanol-fixed cells remained visible under the microscope as single, intact round cells which disappeared upon addition of Drop-seq lysis buffer due to complete cellular lysis (data not shown). Fixation did not induce a microscopically detectable increase in cell doublets.

**Fig. 1:**
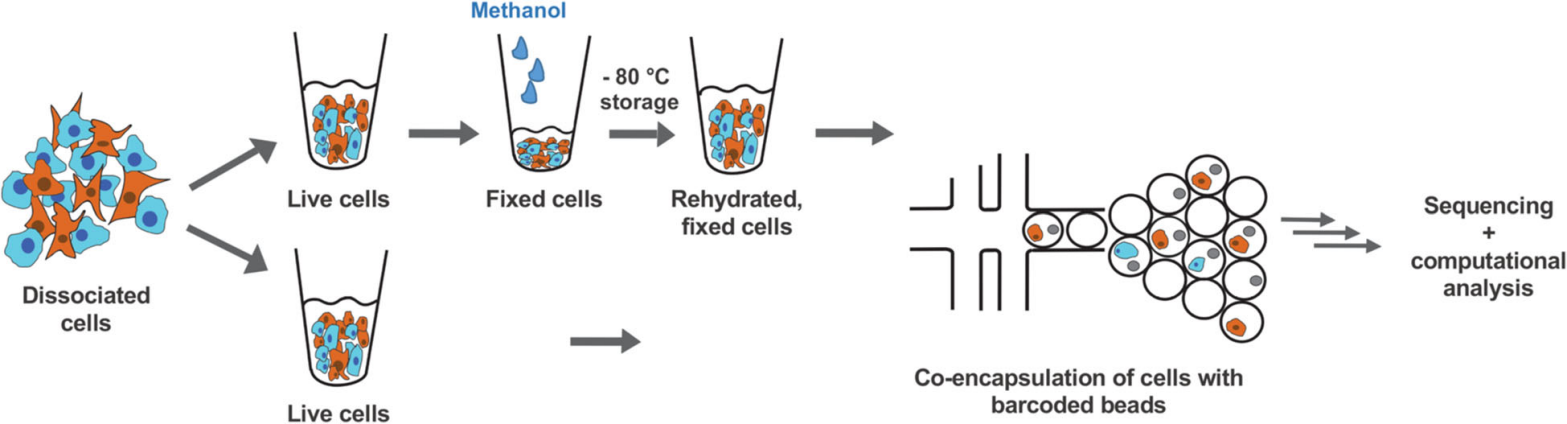
Cell preparation for droplet-based single-cell transcriptional profiling. Schematic of experimental workflow. Cultured human (*HEK*) and mouse (*3T3*) cells were dissociated, mixed and further processed to analyse the transcriptomes of either live or fixed cells by Drop-seq. Washed cells were gently resuspended in 2 volumes of ice-cold PBS, then fixed by adding 8 volumes of ice-cold methanol. Methanol-fixed cells could be stored for up to several weeks at –80 °C. Prior to Drop-seq, cells were washed before passing them through a 35- to 40-μm cell strainer. Cells were then separately encapsulated in droplets together with a single bead in a microfluidic co-flow device and single-cell transcriptomes sequenced in a highly parallel manner. Downstream analysis and systematic quantitative comparisons were subsequently made from separate experiments using live or fixed cellular input material with an R package ('dropbead') that we developed and is freely available for download

**Fig. 2:**
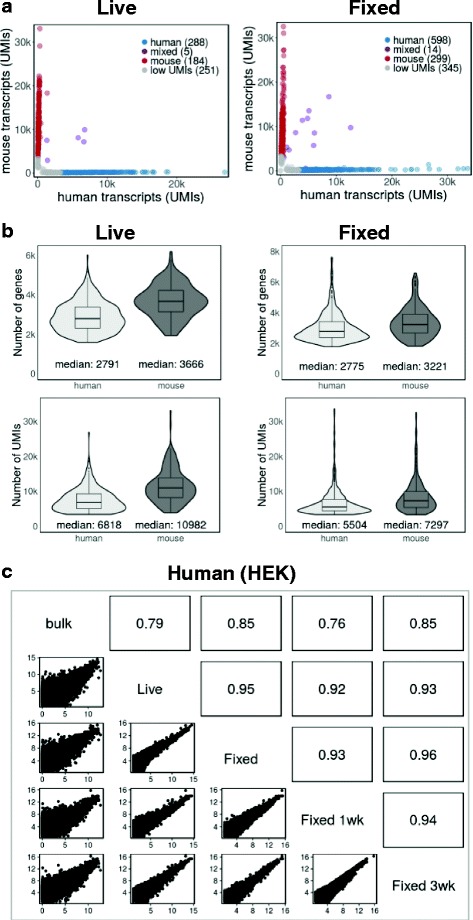
Transcriptome integrities and gene expression levels are preserved in fixed cells. **a** Drop-seq of mixed human and mouse cells (50 cells/μl). Plots show the number of human and mouse transcripts (*UMIs*) associated with a cell (*dot*) identified as human- or mouse-specific (*blue* or *red*, respectively). Cells expressing fewer than 3500 UMIs are *grey*; doublets are *violet*. **b** Distribution and the median of the number of genes and transcripts (UMIs) detected per cell (>3500 UMIs). Libraries were sequenced to a median depth of ~20,500 (*Live*) and ~15,500 (*Fixed*) aligned reads per cell. **c** Gene expression levels from live and fixed cells correlate well. Pairwise correlations between bulk mRNA-seq libraries and Drop-seq single-cell experiments. Non-single cell bulk mRNA-seq data were expressed as reads per kilobase per million (*RPKM*). Drop-seq expression counts were converted to average transcripts per million (*ATPM*) and plotted as log2 (ATPM + 1). *Upper right panels* show Pearson correlation. The overlap (common set) between all five samples is high (17,326 genes). Experiments with live and fixed cells were independently repeated with similar results (unpublished)

To assess the quality of single-cell transcriptomes generated by the Drop-seq procedure after methanol fixation, we used a mixture of cultured human (HEK) and mouse (3T3) cells as performed previously [7]. Both live and fixed cell mixtures were used at a final concentration of 50 cells/μl for Drop-seq runs carried out on the same day, and cDNA libraries were processed in parallel. Figure 1 shows the experimental workflow and Fig. 2 the results of this experiment. We counted the numbers of human and mouse transcripts (UMIs) that were associated with each cell barcode. Both live and fixed cells could be confidently assigned to their species of origin using a threshold of 90% species-specific transcripts (Fig. 2a), suggesting that methanol fixation preserves cell integrity and the species specificity of a cell’s transcriptome. In addition, this experiment showed that fixation did not substantially increase the human/mouse cell doublet rate.

In Drop-seq, cell numbers are selected computationally from the inflection point (’knee’) in a cumulative distribution of reads plotted against the cell barcodes ordered by descending number of reads. Cell barcodes beyond the inflection point are believed to represent ’ambient RNA’ (e.g. contaminating RNA from damaged cells), not cellular transcriptomes [7]. As shown in Additional file 1: Figure S1a, our fixation protocol did not interfere with our ability to computationally select cells.

#### Transcript and gene numbers from live and fixed cells are similar

Figure 2b shows the number and distribution of genes and transcripts (UMIs) in live and fixed cells. Median transcript and gene numbers from fixed cells and their distributions were similar to those of live cells, indicating that methanol fixation did not change the sensitivity of Drop-seq results (Fig. 2b, Additional file 1: Figure S1b). Processing and sequencing a lower number of transcriptomes from the same Drop-seq experiments resulted in higher gene and transcript numbers in both cases (Additional file 1: Figure S1b).

#### Gene expression levels correlate well between live and fixed cells

We treated single-cell transcriptomes as a bulk population and plotted transcript counts from fixed cells against transcript counts from live cells to determine how well they correlate. Figure 2c and Additional file 2: Figure S2c show that gene expression levels from live and fixed cells were highly correlated (*R* ≥ 0.95). Furthermore, transcripts from live and fixed cells against human (HEK) and mouse (3T3) cell bulk mRNA-seq data correlated well (*R* ≥ 0.79; Fig. 2c and Additional file 2: Figure S2c).

Taken together, our data suggest that methanol fixation faithfully preserves single-cell transcriptomes for the Drop-seq procedure.

### Fixed cells can be stored for weeks to give reproducible Drop-seq results

We tested whether fixed cells can be stored, and if so, for how long they can be used for Drop-seq experiments. In order to address this question, we fixed cells and stored them at –80 °C for 1 week or 3 weeks. As shown in Additional files 1 and 2: Figures S1 and S2, single-cell transcriptomes from cells stored for either 1 or 3 weeks performed well in experiments with mixed human and mouse cells. Our results were robust with respect to computational cell selection (Additional file 1: Figure S1a), the ability to assign barcodes to an individual cell’s organism of origin (Additional file 2: Figure S2a) and the median number of genes and transcripts per cell (Additional file 2: Figure S2b).

Finally, gene expression profiles from fixed cells stored for 1 or 3 weeks correlated well with each other and those of cells that were fixed immediately prior to Drop-seq (Fig. 2c and Additional file 2: Figure S2c). They also showed high correlation with bulk mRNA-seq data (Fig. 2c and Additional file 2: Figure S2c). We concluded from these data that fixed cells are stable for several weeks and can be used for Drop-seq experiments without loss in quality.

### Methanol fixation preserves RNA integrity and cytoplasmic RNA content

#### High-quality RNA and cDNA libraries can be prepared from fixed cells

Additional file 1: Figure S1c shows that high-quality, intact total RNA could be extracted from fixed cells after storage in 80% methanol for 20 weeks (RNA was extracted from the same batch of fixed cells that was used to generate the results shown in Additional file 2: Figure S2). Furthermore, Additional file 1: Figure S1 shows BioAnalyzer traces corresponding to all four cDNA libraries analysed in this study: cDNA libraries from methanol-fixed cells appeared indistinguishable from cDNA libraries obtained from live cells (Additional file 1: Figure S1d and unpublished data). Additionally, we confirmed that cDNA libraries from fixed cells did not contain a major 'hidden' peak of low molecular weight fragments normally removed by the library clean-up procedure (Additional file 1: Figure S1e and unpublished data).

#### Mitochondrially encoded transcripts are not strongly elevated in methanol-fixed cells

An increase in the proportion of transcripts from mitochondrial genes (37 mitochondrial DNA-encoded mRNAs) is believed to indicate low-quality cells that are broken or damaged to varying degrees [16]. It is thought that this phenomenon is caused by leakage leading to relative loss of cytoplasmic mRNAs compared to mitochondrially located mRNA transcripts, which are protected by two mitochondrial membranes. As shown in Additional file 1: Figure S1f, we observed less than 10% loss of cellular cytoplasmic mRNA content across all three fixed Drop-seq libraries. Thus, fixation does not seem to cause a major increase in ’low-quality cells’.

Taken together, our data indicate that methanol fixation is able to preserve RNA integrity and subsequent cDNA library generation during the Drop-seq procedure. Our results also show that fixed cells can be stored for prolonged periods up to at least several weeks or months.

### Methanol fixation allows cell type identification in developing *Drosophila* embryos

#### Fixed, primary low RNA content cells from dissociated Drosophila embryos perform well in Drop-seq

**Fig. 3:**
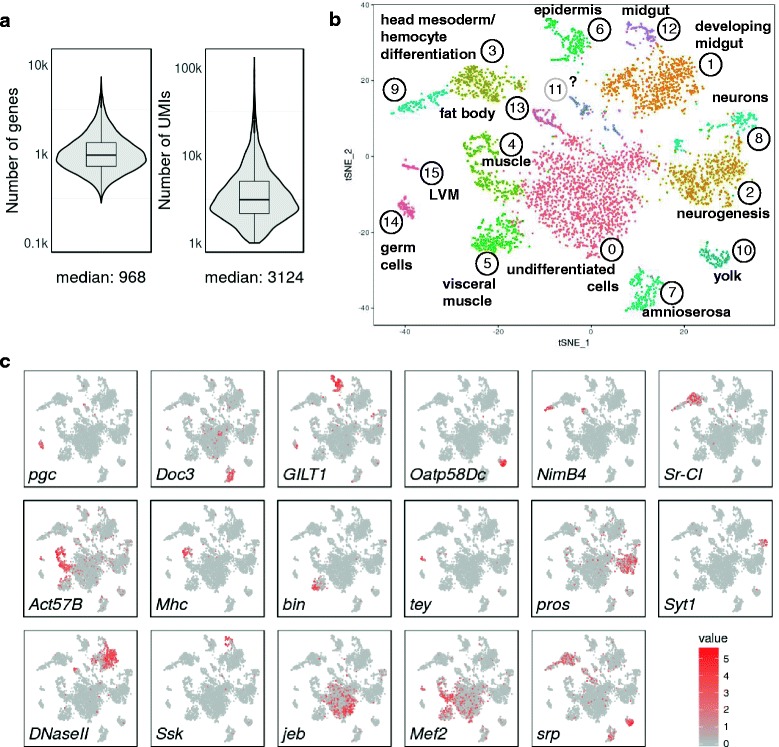
Primary, fixed cells from dissociated *Drosophila* embryos cluster into distinct cell populations. *Drosophila* embryos were collected in 2-h time periods, aged for 6 h, dissociated into single cells and fixed. Drop-seq data correspond to two independent embryo collections, with three and four technical replicates, respectively. Libraries were sequenced to a median depth of ~13,250 aligned reads per cell. Cells expressing fewer than 1000 UMIs were excluded from the analysis. **a** Distribution and the median of the number of genes and transcripts (UMIs) detected per cell in Drop-seq data pooled from seven Drop-seq runs, representing a total of 4873 cells. Note that violin plots are displayed on a log scale. **b** Clustering of 4873 fixed cells into distinct cell populations. The plot shows a two-dimensional representation (tSNE) of global gene expression relationships among all cells. Tissue associations were made by ImaGO term analysis [20] on the 50 most variable genes of each cluster (Additional file 5: Table S1), followed by inspection of publicly accessible RNA in situ staining patterns. *LVM* longitudinal visceral musculature. **c** Marker gene expression in clusters of *Drosophila* embryo cells (see text for explanations). Expression coloured based on normalized expression levels

Primary cells tend to be smaller and contain less RNA than cultured cells, making them harder to analyse by single-cell sequencing [18]. Therefore, we first tested how methanol fixation and subsequent storage performs on primary *Drosophila* cells, which are generally much smaller than most mammalian cell types [19]. Figure 3 and Additional file 3: Figure S3 show the results from seven Drop-seq runs (three and four technical replicates, respectively) performed with two independently collected and processed samples from *Drosophila* embryo pools collected over a 2-h period of time and aged to ensure a rich mixture of differentiating and differentiated cell types (about 75% of developmental stages 10 and 11). The resulting single-cell sequencing data allowed computational selection of cells from ’knee plots’ (Additional file 3: Figure S3a), and cross-correlations of aggregated reads were highly reproducible (*R* ≥ 0.96 across the seven replicates and *R* ≥ 0.82 for comparisons with bulk mRNA-seq samples; Additional files 3 and 4: Figure S3b and Figure S4b). At a sequencing depth of a median of ~13,250 aligned reads per cell (filtering cells with >1000 UMIs and nuclei and cell doublets; see below), we obtained a median of ~1000 genes and ~3000 transcripts (UMIs) per cell (Fig. 3a), indicating that fixation is suitable for primary cells with low RNA content.

#### Methanol fixation allows cell type identification in developing Drosophila embryos

After removing nuclei (characterized by underrepresentation of mitochondrially encoded genes, 2975 cells) and likely cell doublets (2186 cells), we performed principal component analysis (PCA) and two-dimensional (2D) clustering by tSNE using the remaining 4873 cells (Fig. 3b and Additional file 4: Figure S4). Variance was captured in many principal components across distinct embryonic cell populations (Additional file 4: Figure S4a). Clustering analysis revealed numerous cell clusters, most of which could be associated with developing tissues and cell types, based on gene expression profiles (Fig. 3b, Additional file 5: Table S1). Both samples and all Drop-seq runs contributed to the observed clusters, indicating high reproducibility between biological and technical replicates (Additional file 4: Figure S4b).

Tissues were assigned through imaging gene ontology (ImaGO) term analysis [20] of the 50 most variable genes in each cluster (Additional file 5: Table S1) as a first approximation, followed by inspection of publicly available RNA in situ staining patterns [21] of highly variable as well as other known tissue-specific genes. We identified one cluster encompassing an assemblage of undifferentiated cells (cluster 0, marked by genes such as *jelly belly*, *jeb*). Other clusters comprised cell identities corresponding to the germ line and derived from all three germ layers. Known cell-type markers show cluster-specific expression patterns as expected (Fig. 3c) such as in germ cells (*polar granule component*, *pgc*), amnioserosa (T-box transcription factor Dorsocross3, *Doc3*), epidermis (disulphide oxyreductase, *GILT1*) and yolk (*Oatp58Dc*). Clusters 3, 4, 5, 9, 13 and 15 all comprise mesodermally derived cells, albeit in distinct spatial and developmental subpopulations: Clusters 3, 9 and 13 appear to constitute subpopulations of the fat body (pathogen receptor *NimB4*) and head mesoderm, giving rise to differentiating hemocytes/macrophages (scavenger receptor Class C type I, *Sr-CI).* Clusters 4 and 5 include somatic and visceral musculature, but appear to separate the differentiation state of the developing muscle: the more differentiated cells in cluster 4 express contractile machinery (myosin heavy chain, *Mhc*), whereas cluster 5 cells appear less differentiated (*binou*, *bin*). Cluster 15 seems to specifically identify the cells of longitudinal visceral musculature (*tey*). Similarly, ectoderm clusters 2 (*prospero, pros*) and 8 (synaptotagmin 1*, Syt1*) both comprise neurogenic cells, but cluster 2 cells appear to be developmentally less differentiated. Accordingly, biological process gene ontology (GO) term enrichment for cluster 2 terms was more generally linked to nervous system development, whereas cluster 8 was linked to synaptic signalling and other terms indicating a functioning nervous system. Clusters 1 (*DNAse II*) and 12 (Snakeskin*, Ssk*) are both endodermal cell populations, but while cluster 1 primarily constitutes the mid- and hindgut primordium, cluster 12 contains more differentiated, functional cells of the gut. Lastly, *myocyte enhancing factor* (*Mef2*) and *serpent* (*srp*) are two examples of transcription factors which are expressed in distinct mesoderm-derived clusters, as expected at this stage of development.

### Sorted, fixed mouse brain cells allow identification of distinct neural and non-neural cell types

**Fig. 4:**
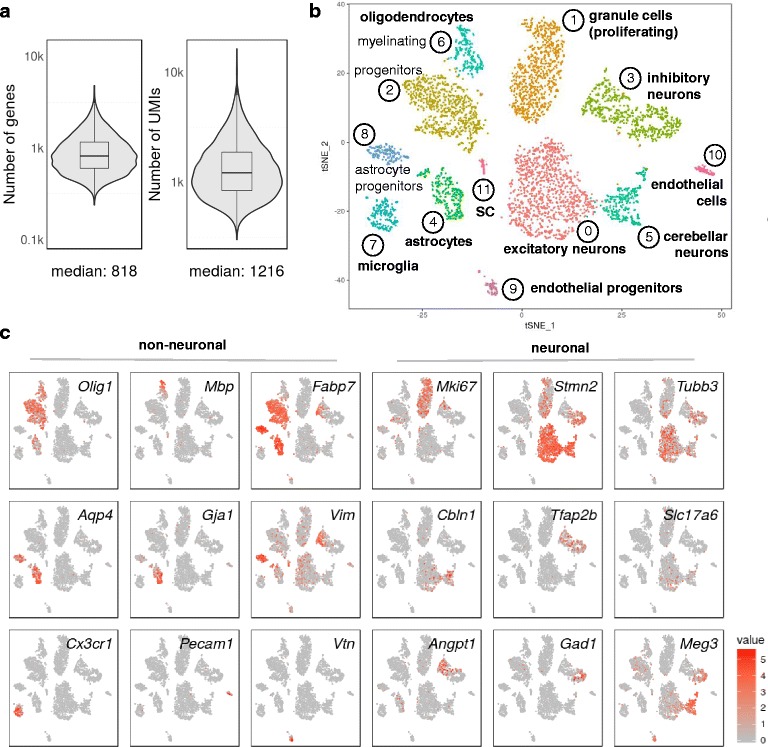
Sorted, fixed mouse brain cells allow identification of distinct neural and non-neural cell types. Hindbrains and cerebellum from newborn mice were microdissected and dissociated, and cells were sorted by FACS into methanol and stored. Drop-seq data correspond to two independent biological replicates. Libraries were sequenced to a median depth of ~7100 reads per cell. **a** Distribution and the median of the number of genes detected per cell (>300 UMIs) in Drop-seq data pooled from two Drop-seq runs, representing 4366 cells. Note that violin plots are displayed on a log scale. **b** Clustering of 4366 fixed cells into distinct cell populations marked by colour (Additional file 8: Table S2). The plot shows a two-dimensional representation (tSNE) of global gene expression relationships among all cells. Tissue associations of cell clusters were identified by assessing the 50 most variable genes in each cluster and confirmed by inspection of publicly accessible images of RNA in situ hybridizations. **c** Known marker gene expression in clusters of brain cells (see text for explanation). Expression coloured based on normalized expression levels

In order to address the question of whether methanol fixation can be used in conjunction with fluorescence-activated cell so rting (FACS), we dissected hindbrain and cerebellum from newborn mice, dissociated the cells and sorted live, propidium iodide-negative cells directly into ice-cold methanol. We expected to obtain a mixture of neurons, glia and non-neuronal cell types for further analysis. Two independent biological replicates allowed computational cell selection from ’knee plots’ (Additional file 6: Figure S5a, b) and were highly reproducible (*R* = 0.95; *R* ≥ 0.8 for comparisons with independently prepared bulk mRNA-seq data). After combining the data and removing low-quality cells (expressing <300 UMIs) as well as cell doublets (1127 cells), we obtained a median of ~800 genes and ~1200 transcripts (UMIs) per cell for the remaining 4366 cells at a shallow sequencing depth of a median of ~7100 aligned reads per cell; Fig. 4a. PCA revealed variance captured in many principal components (Additional file 7: Figure S6a), and 2D representation by tSNE produced 12 clusters, all of which contained readily identifiable cell types (Fig. 4b; Additional file 8: Table S2). Both biological replicates contributed to all observed clusters (Additional file 7: Figure S6b).

Cell populations were identified through their gene expression signatures (Fig. 4c; Additional file 8: Table S2) and encompassed neurons and glial cells. We identified different neuronal cell types such as proliferating granule cells (proliferation marker *Mki67*; neuronal marker stathmin-like 2, *Stmn2*), excitatory neurons (glutamatergic neuronal marker *Slc17a6*/*VGLUT2* and neuronal markers *Stmn2* and tubulin beta 3, *Tubb3*), inhibitory neurons (GABAergic neuronal markers GAD67, *Gad1* and VGAT, *Slc32a﻿1*/; *Tubb3*; transcription factor AP2 beta, *Tfap2b*) and cerebellar neurons (Cerebellin, *Cbln1*; lncRNA *Meg3*; *Stmn2*). Among the glial cells, we identified oligodendrocyte progenitors (oligodendrocyte transcription factor 1, *Olig1*; fatty acid binding protein 7, *Fabp7*), myelinating oligodendrocytes (myelin basic protein, *Mbp*; *Olig1*), microglia (chemokine receptor *Cx3cr1*) and astrocytes (gap junction protein alpha 1, *Gja1*; aquaporin 4, *Aqp4*; *Fabp7*) as well as astrocyte/neuronal progenitors (vimentin, *Vim*; *Aqp4*, *Fabp7*). We also identified a subtype of myelinating glia (cluster 11), which expressed myelin protein zero (*Mpz*), probably Schwann cells from cranial nerves entering the hindbrain (Additional file 7: Figure S6c). In addition, we found non-neural cell types such as endothelial cells expressing platelet/endothelial adhesion molecule 1 (*Pecam1*) and endothelial progenitors (vitronection, *Vtn*). Markers were confirmed to be expressed in newborn hindbrain and cerebellum by inspecting RNA in situ hybridization images in publicly available databases [22, 23].

Together, our data demonstrate that methanol can be used to fix and store primary cells for Droplet-based sequencing, including low input RNA cells such as differentiating embryonic *Drosophila* cells and a wide variety of mammalian brain cells, including neuronal subtypes.

## Discussion

Few studies so far have explicitly dealt with cell preservation protocols for single-cell sequencing. One study uses cryopreservation followed by flow cytometry to sort single cells for subsequent processing [24]. While cryopreservation is compatible with droplet-sequencing in principle [18], it remains to be determined how well Drop-seq will perform with recently thawed cells that may be fragile and prone to die. Another study describes fixation of cells by cross-linking with formaldehyde [25], followed by reverse cross-linking (breakage of methylene bridges between protein and RNA molecules with heat). Cross-linking induces chemical modifications that inhibit poly(dT) annealing, reverse transcriptase and cDNA synthesis, making cross-link reversal necessary [10]. However, reversal of cross-linking is often inefficient, leaves adducts and can be expected to result in high loss of available RNA molecules in a non-uniform manner.

We have shown here that a simple methanol fixation of tissue culture cells does not lead to significant RNA loss or degradation and is easily compatible with the established Drop-seq single-cell sequencing protocol and workflow. It is possible to store single-cell suspensions for prolonged times at low temperatures and, therefore, to separate the sample preparation phase in time or location from the actual droplet-sequencing procedure. In addition to the cell culture lines used in this study (human HEK cells, mouse NIH/3T3 cells), we have successfully applied our methanol fixation protocol to a variety of other cell lines or cultured cells (HeLa cells, mouse cycling and non-cycling pre-B and primary mouse lymphoma cells).

Beyond cultured cells, which in many instances contain more RNA than oftentimes smaller, primary cells [18], Drop-seq can be successfully performed with fixed cells from complex tissues such as dissociated, later stage *Drosophila* embryos and mouse brain, as shown in this study. Single-cell sequencing data from individual runs of primary tissues from both organisms yielded highly reproducible results. Even though we applied only a relatively shallow sequencing depth (a median of ~7100 aligned reads per cell) to our mouse brain sample, the single-cell sequencing data allowed identification of diverse cell types and subpopulations of cells, including subcluster differentiation and developmental trajectories (Fig. 4c) [26, 27]. For example, a large cluster of inhibitory neurons (marked by *Gad1*, *Tfap2b*; cluster 3 in Fig. 4b, c) contained less mature, still dividing cells in the left part (marked by *Vim*, *Mki67* and *Angpt1*) and more mature neurons in the right part (*Tubb3*, *Stmn2*, *Meg3*). Furthermore, in the oligodendrocyte clusters (2 and 6 in Fig. 4b), more mature, myelinating oligodendrocytes clustered to the upper left of cluster 6 (marked by *Mbp*, *Olig1*), newly formed oligodendrocytes in the middle of cluster 2 (*Olig1*, *Fabp7*) and still dividing progenitors to the lower part of cluster 2 (marked by *Mki67*, *Fabp7*, *Olig1*).

We also found that single-cell sequencing data from methanol-fixed cells were of sufficient quality to carry out spatial mapping and 3D reconstruction of a virtual *Drosophila* embryo at the onset of gastrulation [14]. Methanol fixation allowed us to prepare and store cells from carefully staged, early embryos incrementally in small batches.

However, methanol fixation may not work in all circumstances, for all tissues or cell types. Successful fixation may be challenging, especially for tissues with a high content in proteases and RNAses such as pancreas, gall bladder, skin or lymphatic and immune tissues. In support of this notion, we observed a failure to generate Drop-seq cDNA libraries and to isolate intact RNA from fixed cells of a mouse lymphoma ex vivo (unpublished results). For these types of tissues, it will be important to determine at which step RNA degradation occurs, before or after fixation. Modifications to the fixation protocol such as addition of RNAse inhibitor during the rehydration step (as used in our experiments with primary cells) may remedy these problems. It also remains to be determined whether methanol fixation is compatible with the ‘InDrop’ protocol [8], another, recently developed droplet-based sequencing approach that involves a different detergent for cell lysis and cDNA library construction. A recent study that uses combinatorial indexing to transcriptionally profile large numbers of single cells relies on methanol fixation [28], suggesting that alcohol-based fixation may be compatible with a wider range of single-cell sequencing approaches.

## Conclusions

The availability of a simple cell fixation protocol will open up many previously inaccessible experimental avenues for droplet-based single-cell transcriptomics. Fixation and preservation of cells at an early stage of preparation removes bias and technical variation, prevents cell stress or unintended ageing during the experiment and facilitates systematic assessment of experimental parameters. Cell fixation may also significantly ease the logistic coordination of large-scale experiments. In a variety of situations, fixation may be the only solution to being able to process and provide cellular input material: examples are rare cells which cannot be obtained in one experimental session [14], clinical specimens which require transportation or cells that are hard to isolate and require extensive upstream processing, such as tissue dissociation followed by flow cytometry. In summary, we expect that the methanol-based cell fixation procedure presented here will greatly stimulate high-throughput single-cell sequencing studies in diverse areas.

## Additional files

Additional file 1: Figure S1. Computational cell selection and RNA, cDNA library and cell quality. Related to Fig. 2. (a) Identification of cell barcodes associated with single-cell transcriptomes in a pool of amplified single-cell libraries. Drop-seq involves Poisson-limited dilution of cells, implying that most beads (> 95%) are only exposed to ambient RNA. To identify the cell barcodes associated with cellular transcriptomes, cell barcodes are plotted in decreasing order of reads against the cumulative fraction of reads. The inflection point (*red line*) indicates the number of cells; human-mouse cell doublets were removed computationally. Note that sample 'Fixed 1 week' has fewer cells, because only a fraction of barcoded beads was used for library preparation. (b) A subset of cells from the experiment depicted in Fig. 2 (Live: 99 human, 44 mouse cells; Fixed: 253 human, 90 mouse cells) was sequenced at a higher median depth of ~104,106 and ~53,500 aligned reads per cell. Note that the live sample appears to have more genes and UMIs, because fewer cells were sequenced, resulting in more reads per cell. (c)–(e) Bioanalyzer traces. (c) High-quality RNA could be extracted from rehydrated cells that were fixed and stored for 20 weeks. (d) Fixation and storage does not change the fragment size distribution of Drop-seq cDNA libraries. Libraries were purified with 0.6× Solid Phase Reversible Immobilization (*SPRI*) beads. (e) Parallel control purification of the cDNA library ’Fixed 3 weeks’ with 0.6× (fragments above 500 bp; *upper panel*) or 1.8× SPRI beads (all fragments; *lower panel*) did not reveal a major peak corresponding to small molecular weight fragments indicative of low-quality RNA input cells. (f) Plot depicting the percentage of reads mapping to non-mitochondrially encoded genes. Stressed or broken cells lose non-mitochondrially encoded, cytoplasmically localized mRNAs [16]. Loss of cytoplasmic reads in fixed cells was < 10%. (PDF 481 kb)

Additional file 2: Figure S2. Fixed cell samples can be stored for weeks to give reproducible results. Related to Fig. 2. (a), (b) Drop-seq of mixed human and mouse cells (50 cells/μl), corresponding to a biological replicate of the experiment shown in Fig. 2. Libraries were sequenced to a median depth of ~142,400 (Fixed 1 week) or ~28,500 (Fixed 3 weeks) aligned reads per cell. (a) Plots show the number of human and mouse transcripts (*UMIs*) associated with a cell (*dot*) identified as human- or mouse-specific (*blue or red*, respectively). Cells expressing fewer than 3500 UMIs are *grey*. Both Drop-seq experiments yielded single-cell transcriptomes that allowed clear species separation and a low percentage of cell doublets. (b) Distribution and the median of the number of genes and transcripts (UMIs) detected per cell expressing more than 3500 UMIs. (c) Gene expression levels from live and fixed cells correlate well. Pairwise correlations between bulk mRNA-seq libraries and Drop-seq single-cell experiments for cells expressing more than 3500 UMIs. Non-single cell bulk mRNA-seq data are shown as reads per kilobase per million (*RPKM*). Drop-seq expression counts were converted to average transcripts per million (*ATPM*) and plotted as log2 (ATPM + 1). *Upper right panel* depicts Pearson correlations. The intersection (common set) of genes between all samples was high (~17,000 genes). (PDF 228 kb)

Additional file 3: Figure S3. Single-cell data from *Drosophila* embryos are reproducible and correlate well with bulk mRNA-seq data. Related to Fig. 3. (a) Identification of cell barcodes associated with single-cell transcriptomes for single-cell libraries from *Drosophila* embryos, a complex primary tissue harbouring small, low RNA content cells. (For methods details, see Additional file 1: Figure S1a.) Four of seven replicates are shown. (b) Correlations between gene expression measurements from bulk mRNA-seq and seven Drop-seq runs with methanol-fixed single cells (expressing >1000 UMIs). Cells were from two independent biological samples representing dissociated *Drosophila* embryos (75% stages 10 and 11). Bulk mRNA-seq data were generated with total RNA extracted directly from whole, intact, live embryos. (Sample 1: rep 1, 2, 7 and bulk 1; sample 2: rep 3–6 and bulk 2). Non-single cell bulk mRNA-seq data were expressed as reads per kilobase per million (*RPKM*). Drop-seq expression counts were converted to average transcripts per million (*ATPM*) and plotted as log2 (ATPM + 1). *Upper right panel* depicts Pearson correlations. The intersection (common set) of genes between all samples was high (~10,000 genes). (PDF 162 kb)

Additional file 4: Figure S4. Variance in single-cell data from *Drosophila* embryos and 2D cluster representations of replicates. Related to Fig. 3. (a) Plots of principal components 1–30 of the 4873 cell transcriptomes show variance captured in many principal components. Colors correspond to tSNE plot in Fig. 3b. (b) 2D representation of experimental replicates in each cell population. tSNE plot from Fig. 3b with cells now coloured by experimental Drop-seq replicate (*left*) or biological replicate sample (*right*). Clusters are formed by cells from many Drop-seq different runs (*left*) and from both samples (*right*). The relatively more homogenous composition of cluster 8 (neurons) and 15 (LVM) is consistent with a higher proportion of embryos of later stages in sample 2. (PDF 376 kb)

Additional file 5: Table S1. Top 50 marker genes expressed in 4873 fixed, primary cells from *Drosophila* embryos. Related to Fig. 3. Tables S1 and S2 contain the top 50 marker genes per cluster, provided by Seurat's function 'FindAllMarkers' [17]. We additionally ordered them per cluster in decreasing log2-fold change (log2FC). The log2FC was computed for a given gene by dividing its average normalized expression for a given cluster over the average normalized expression in the rest of the clusters and taking the logarithm of the fold change. (XLSX 214 kb)

Additional file 6: Figure S5. Single-cell data from mouse hindbrain are reproducible and correlate well with bulk mRNA-seq data. Related to Fig. 4. (a) Identification of cell barcodes associated with single-cell transcriptomes for single-cell libraries from FACS-sorted, fixed mouse hindbrain cells. (For methods details, see Additional file 1: Figure S1). (b) Correlations between gene expression measurements from independent Drop-seq experiments with FACS-sorted methanol-fixed single cells (expressing >300 UMIs). Cells were from independent biological samples, representing dissected, dissociated mouse hindbrains and cerebellum from newborn mice. Bulk mRNA-seq data were generated with total RNA extracted from cells after FACS and fixation. Non-single cell bulk mRNA-seq data were expressed as reads per kilobase per million (*RPKM*). Drop-seq expression counts were converted to average transcripts per million (*ATPM*) and plotted as log2 (ATPM + 1). *Upper right panel* depicts Pearson correlations. The intersection (common set) of genes between samples was ~17,000 genes. (PDF 68 kb)

Additional file 7: Figure S6. Variance in single-cell data from newborn mouse hindbrain and cerebellum and 2D cluster representation of replicates. Related to Fig. 4. (a) Plots of principal components 1–18 of the 4366 cell transcriptomes show variance in many principal components. Colors correspond to tSNE plot in Fig. 4b. (b) 2D representation of experimental replicates in each cell population. tSNE plot from Fig. 4b with each cell now coloured by experimental replicate. Note that cells from the two biological replicates are unevenly represented in the different clusters, likely reflecting dissection differences and varying proportions of hindbrain to cerebellar tissue. (c) We identified a subtype of myelinating glia, probably Schwann cells from cranial nerves entering the hindbrain (cluster 11, Fig. 4b). These cells express myelin protein zero (*Mpz*) and other genes for myelin formation (proteolipid protein 1, *Plp1*) and *Mbp* (Fig. 4b) but do not express oligodendrocyte markers such as *Bcas1* or *Olig1* (Fig. 4b). (PDF 255 kb)

Additional file 8: Table S2. Top 50 marker genes expressed in 4366 sorted, fixed cells from mouse hindbrain and cerebellum. For explanations, see legend to Table S1. Related to Fig. 4. (XLSX 196 kb)
