# Supplementary figures and images for: Cell fixation and preservation for droplet-based single-cell transcriptomics
Source: BMC Biol. 2017 May 19;15:44. doi: 10.1186/s12915-017-0383-5 (PMC5438562; doi:10.1186/s12915-017-0383-5)

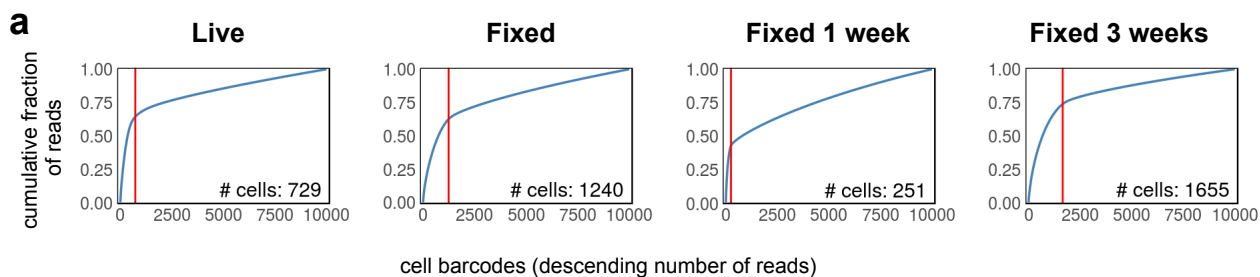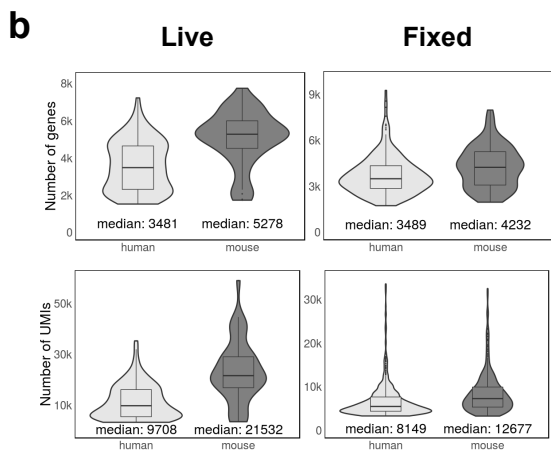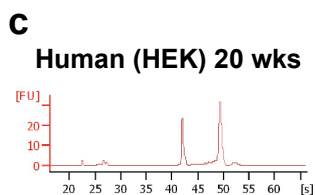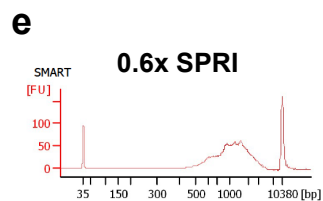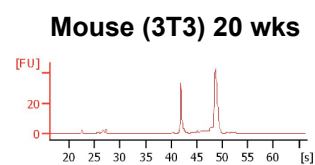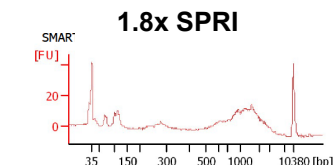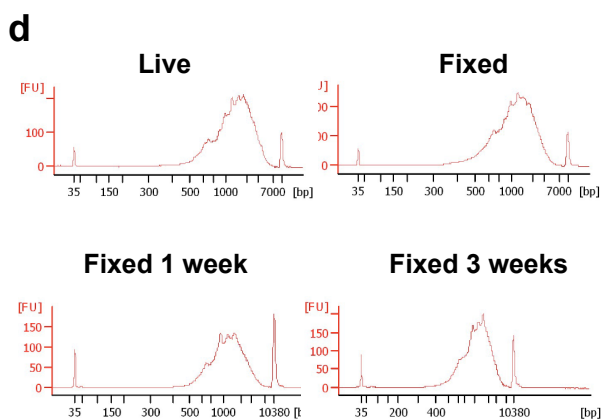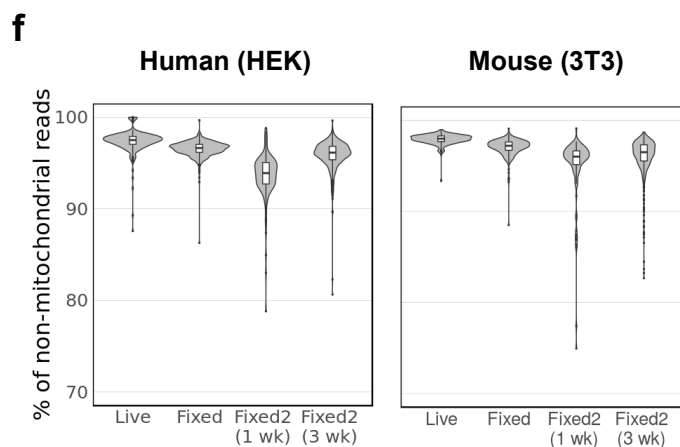

Supplement: Supplementary file 1 — Computational cell selection and RNA, cDNA library and cell quality. Related to Fig. 2. (a) Identification of cell barcodes associated with single-cell transcriptomes in a pool of amplified single-cell libraries. Drop-seq involves Poisson-limited dilution of cells, implying that most beads (> 95%) are only exposed to ambient RNA. To identify the cell barcodes associated with cellular transcriptomes, cell barcodes are plotted in decreasing order of reads against the cumulative fraction of reads. The inflection point (red line) indicates the number of cells; human-mouse cell doublets were removed computationally. Note that sample 'Fixed 1 week' has fewer cells, because only a fraction of barcoded beads was used for library preparation. (b) A subset of cells from the experiment depicted in Fig. 2 (Live: 99 human, 44 mouse cells; Fixed: 253 human, 90 mouse cells) was sequenced at a higher median depth of ~104,106 and ~53,500 aligned reads per cell. Note that the live sample appears to have more genes and UMIs, because fewer cells were sequenced, resulting in more reads per cell. (c)–(e) Bioanalyzer traces. (c) High-quality RNA could be extracted from rehydrated cells that were fixed and stored for 20 weeks. (d) Fixation and storage does not change the fragment size distribution of Drop-seq cDNA libraries. Libraries were purified with 0.6× Solid Phase Reversible Immobilization (SPRI) beads. (e) Parallel control purification of the cDNA library ’Fixed 3 weeks’ with 0.6× (fragments above 500 bp; upper panel) or 1.8× SPRI beads (all fragments; lower panel) did not reveal a major peak corresponding to small molecular weight fragments indicative of low-quality RNA input cells. (f) Plot depicting the percentage of reads mapping to non-mitochondrially encoded genes. Stressed or broken cells lose non-mitochondrially encoded, cytoplasmically localized mRNAs [16]. Loss of cytoplasmic reads in fixed cells was < 10%. (PDF 481 kb) [file 12915_2017_383_MOESM1_ESM.pdf]

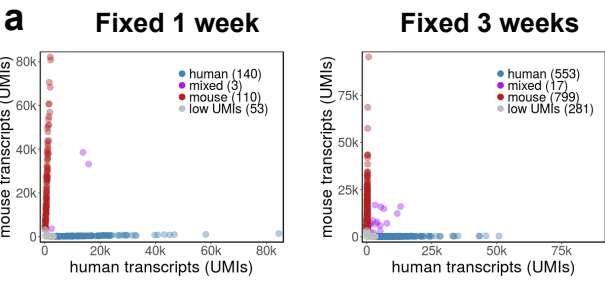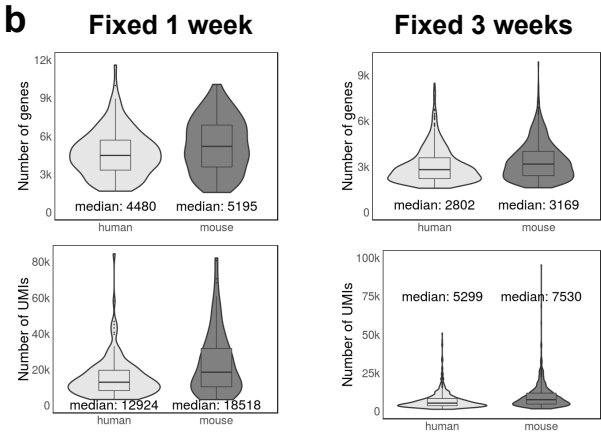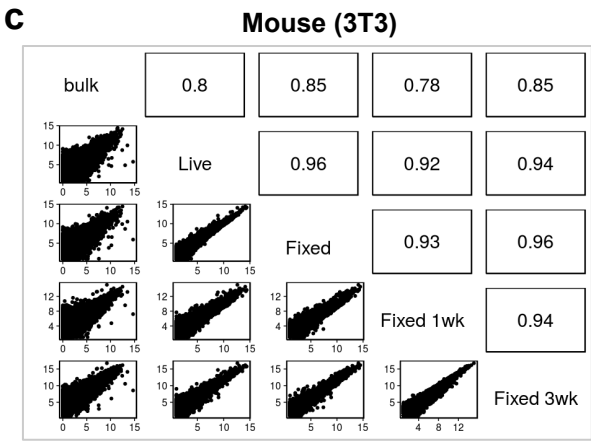

Supplement: Supplementary file 2 — Fixed cell samples can be stored for weeks to give reproducible results. Related to Fig. 2. (a), (b) Drop-seq of mixed human and mouse cells (50 cells/μl), corresponding to a biological replicate of the experiment shown in Fig. 2. Libraries were sequenced to a median depth of ~142,400 (Fixed 1 week) or ~28,500 (Fixed 3 weeks) aligned reads per cell. (a) Plots show the number of human and mouse transcripts (UMIs) associated with a cell (dot) identified as human- or mouse-specific (blue or red, respectively). Cells expressing fewer than 3500 UMIs are grey. Both Drop-seq experiments yielded single-cell transcriptomes that allowed clear species separation and a low percentage of cell doublets. (b) Distribution and the median of the number of genes and transcripts (UMIs) detected per cell expressing more than 3500 UMIs. (c) Gene expression levels from live and fixed cells correlate well. Pairwise correlations between bulk mRNA-seq libraries and Drop-seq single-cell experiments for cells expressing more than 3500 UMIs. Non-single cell bulk mRNA-seq data are shown as reads per kilobase per million (RPKM). Drop-seq expression counts were converted to average transcripts per million (ATPM) and plotted as log2 (ATPM + 1). Upper right panel depicts Pearson correlations. The intersection (common set) of genes between all samples was high (~17,000 genes). (PDF 228 kb) [file 12915_2017_383_MOESM2_ESM.pdf]

**a**

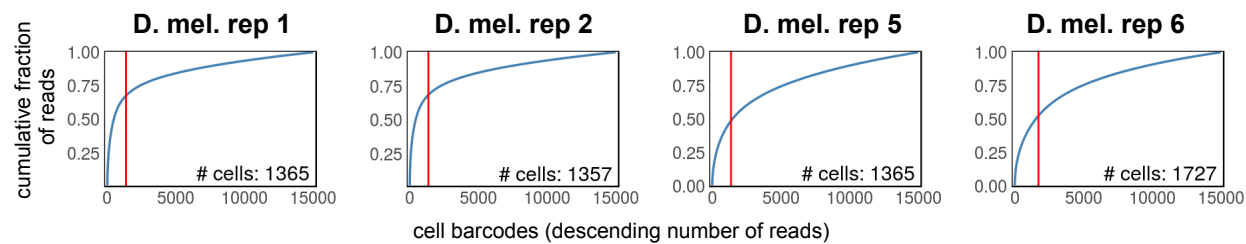

**b**

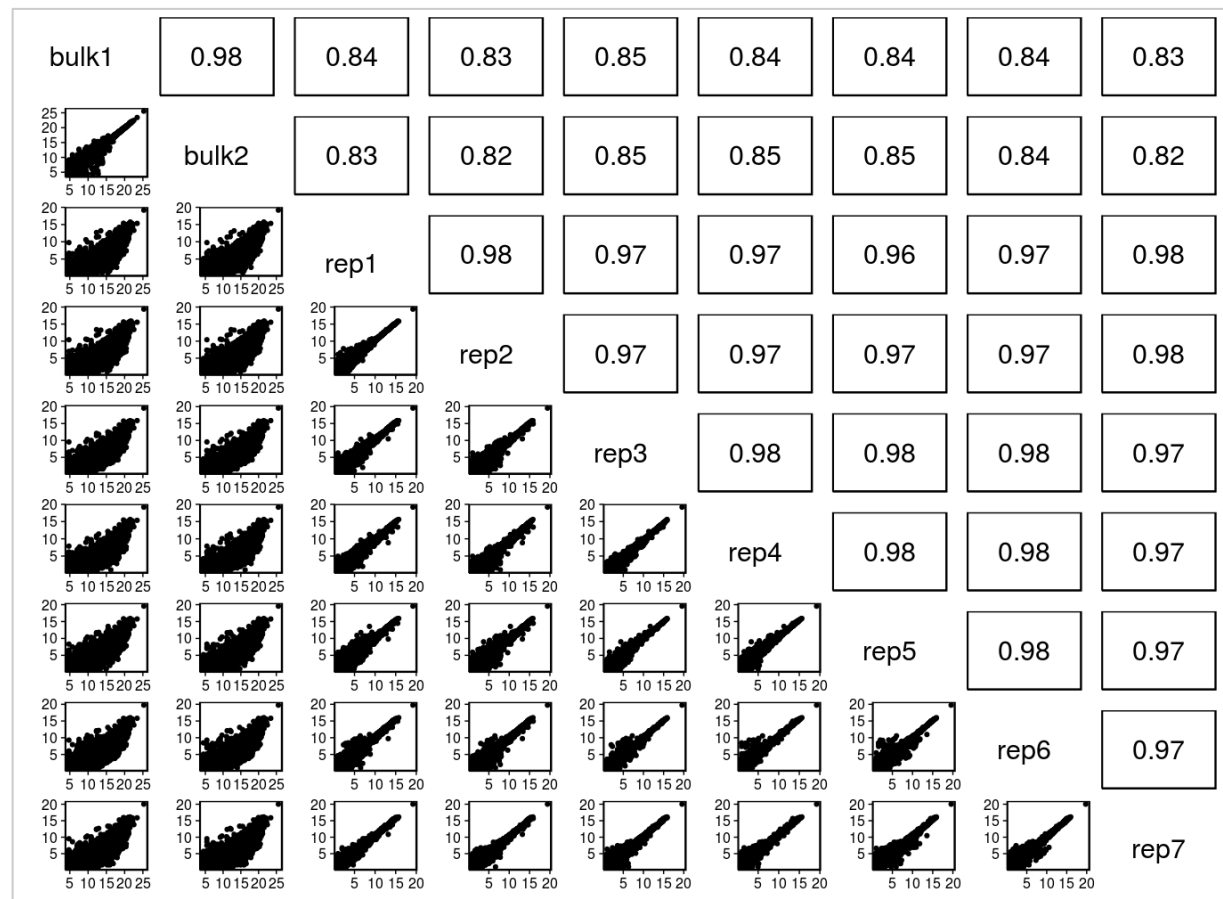

Supplement: Supplementary file 3 — Single-cell data from Drosophila embryos are reproducible and correlate well with bulk mRNA-seq data. Related to Fig. 3. (a) Identification of cell barcodes associated with single-cell transcriptomes for single-cell libraries from Drosophila embryos, a complex primary tissue harbouring small, low RNA content cells. (For methods details, see Additional file 1: Figure S1a.) Four of seven replicates are shown. (b) Correlations between gene expression measurements from bulk mRNA-seq and seven Drop-seq runs with methanol-fixed single cells (expressing >1000 UMIs). Cells were from two independent biological samples representing dissociated Drosophila embryos (75% stages 10 and 11). Bulk mRNA-seq data were generated with total RNA extracted directly from whole, intact, live embryos. (Sample 1: rep 1, 2, 7 and bulk 1; sample 2: rep 3–6 and bulk 2). Non-single cell bulk mRNA-seq data were expressed as reads per kilobase per million (RPKM). Drop-seq expression counts were converted to average transcripts per million (ATPM) and plotted as log2 (ATPM + 1). Upper right panel depicts Pearson correlations. The intersection (common set) of genes between all samples was high (~10,000 genes). (PDF 162 kb) [file 12915_2017_383_MOESM3_ESM.pdf]

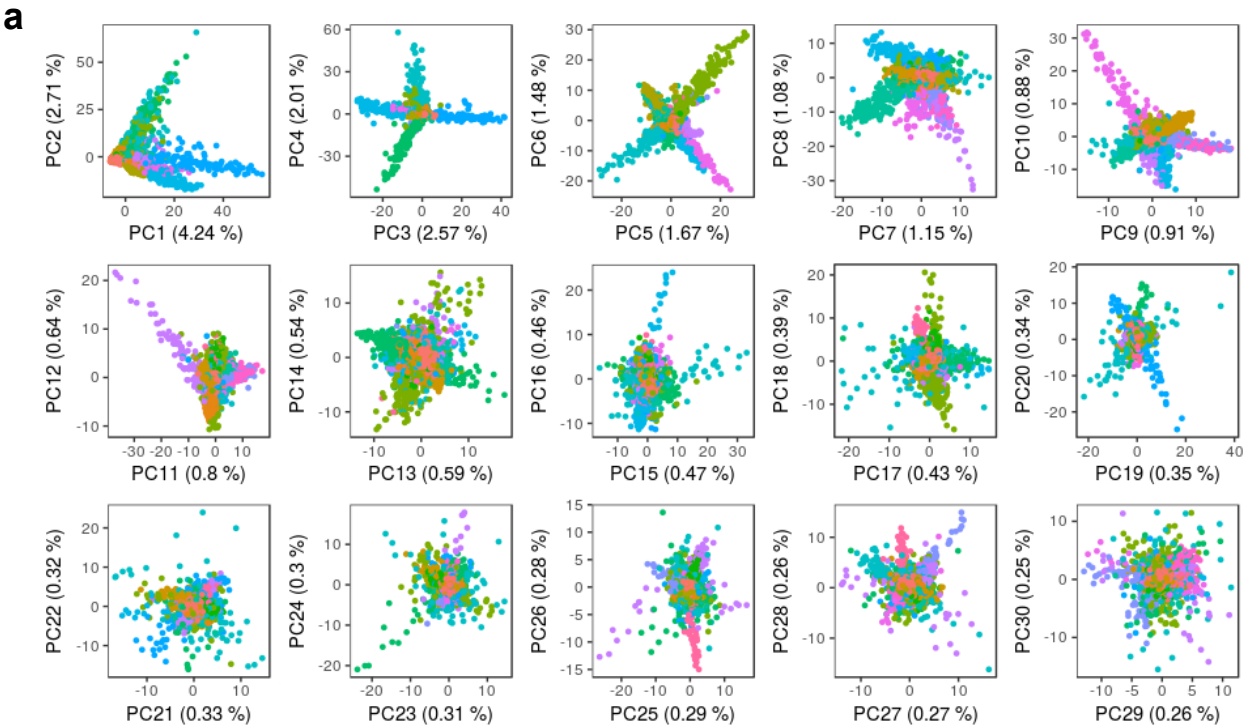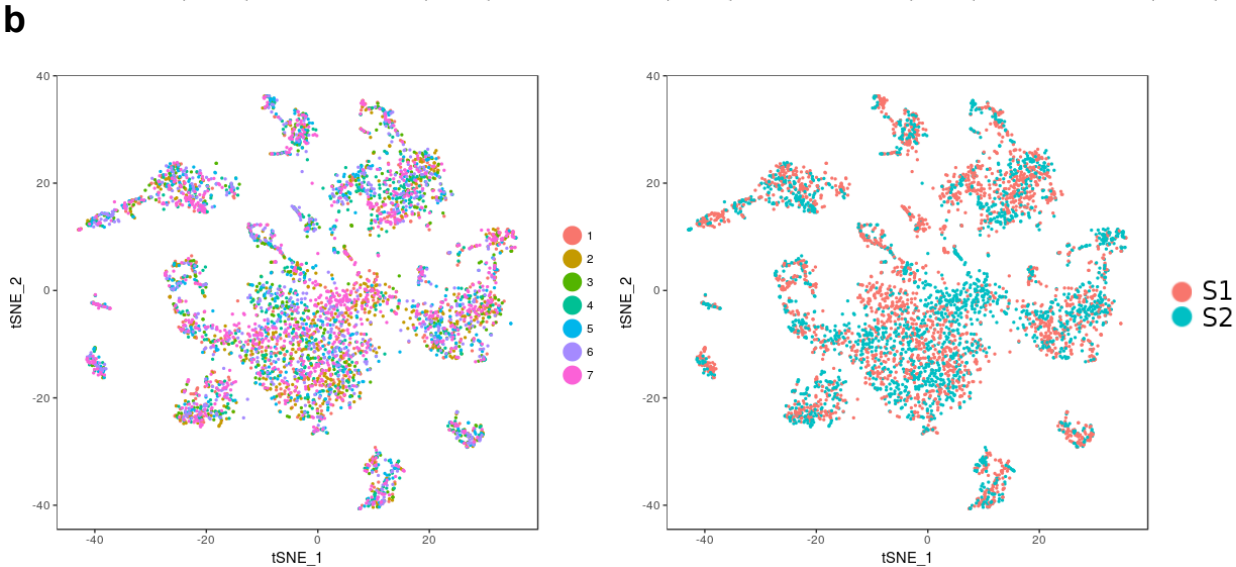

Supplement: Supplementary file 4 — Variance in single-cell data from Drosophila embryos and 2D cluster representations of replicates. Related to Fig. 3. (a) Plots of principal components 1–30 of the 4873 cell transcriptomes show variance captured in many principal components. Colors correspond to tSNE plot in Fig. 3b. (b) 2D representation of experimental replicates in each cell population. tSNE plot from Fig. 3b with cells now coloured by experimental Drop-seq replicate (left) or biological replicate sample (right). Clusters are formed by cells from many Drop-seq different runs (left) and from both samples (right). The relatively more homogenous composition of cluster 8 (neurons) and 15 (LVM) is consistent with a higher proportion of embryos of later stages in sample 2. (PDF 376 kb) [file 12915_2017_383_MOESM4_ESM.pdf]

**a**

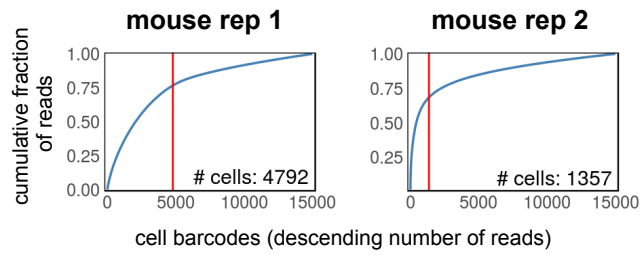

**b**

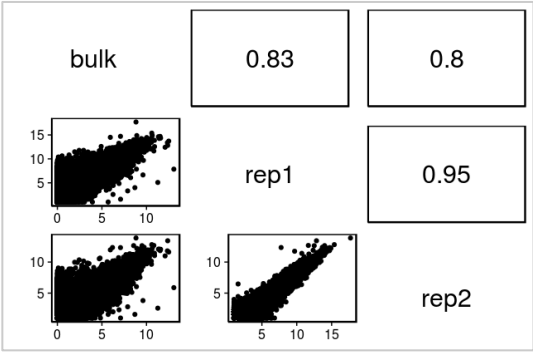

Supplement: Supplementary file 6 — Single-cell data from mouse hindbrain are reproducible and correlate well with bulk mRNA-seq data. Related to Fig. 4. (a) Identification of cell barcodes associated with single-cell transcriptomes for single-cell libraries from FACS-sorted, fixed mouse hindbrain cells. (For methods details, see Additional file 1: Figure S1). (b) Correlations between gene expression measurements from independent Drop-seq experiments with FACS-sorted methanol-fixed single cells (expressing >300 UMIs). Cells were from independent biological samples, representing dissected, dissociated mouse hindbrains and cerebellum from newborn mice. Bulk mRNA-seq data were generated with total RNA extracted from cells after FACS and fixation. Non-single cell bulk mRNA-seq data were expressed as reads per kilobase per million (RPKM). Drop-seq expression counts were converted to average transcripts per million (ATPM) and plotted as log2 (ATPM + 1). Upper right panel depicts Pearson correlations. The intersection (common set) of genes between samples was ~17,000 genes. (PDF 68 kb) [file 12915_2017_383_MOESM6_ESM.pdf]

**a**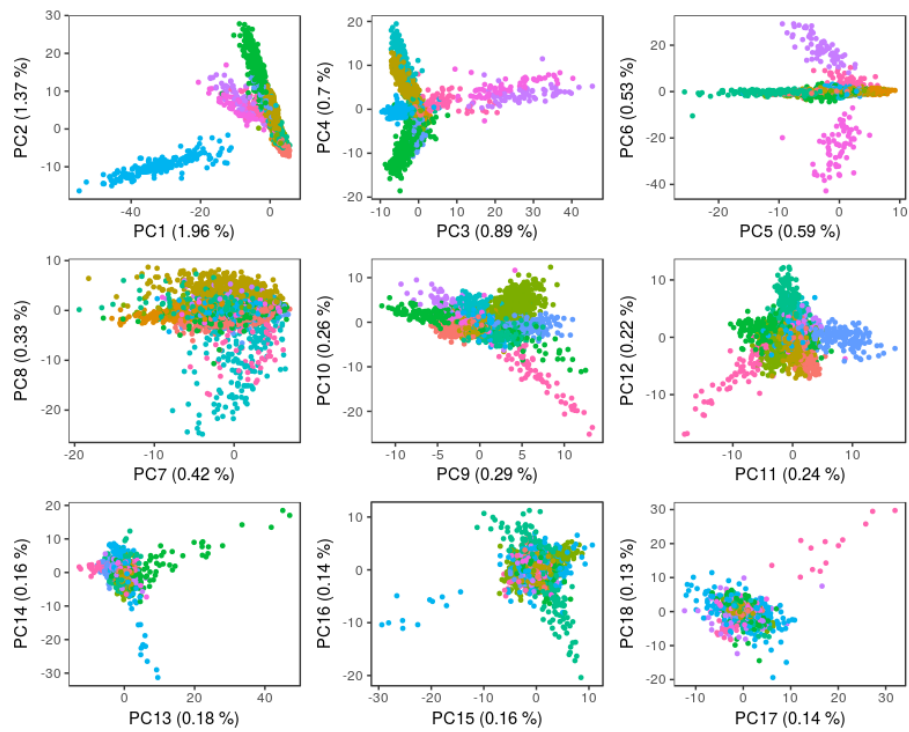**b**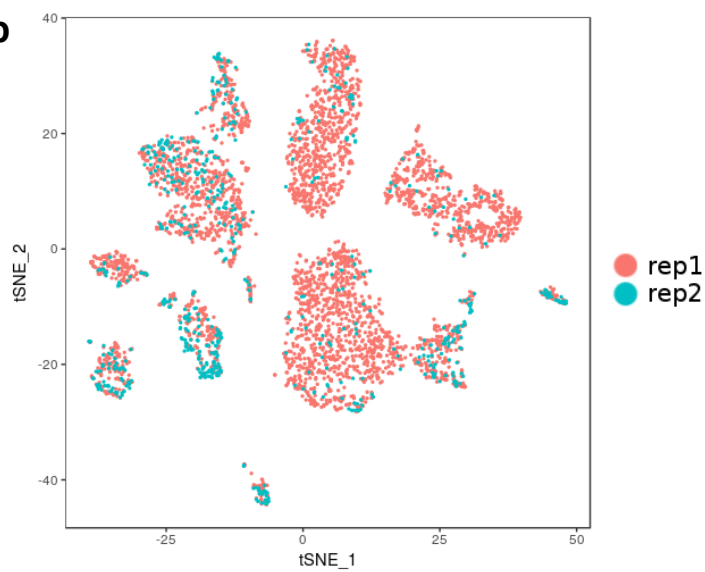**c**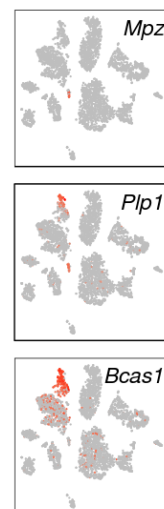

Supplement: Supplementary file 7 — Variance in single-cell data from newborn mouse hindbrain and cerebellum and 2D cluster representation of replicates. Related to Fig. 4. (a) Plots of principal components 1–18 of the 4366 cell transcriptomes show variance in many principal components. Colors correspond to tSNE plot in Fig. 4b. (b) 2D representation of experimental replicates in each cell population. tSNE plot from Fig. 4b with each cell now coloured by experimental replicate. Note that cells from the two biological replicates are unevenly represented in the different clusters, likely reflecting dissection differences and varying proportions of hindbrain to cerebellar tissue. (c) We identified a subtype of myelinating glia, probably Schwann cells from cranial nerves entering the hindbrain (cluster 11, Fig. 4b). These cells express myelin protein zero (Mpz) and other genes for myelin formation (proteolipid protein 1, Plp1) and Mbp (Fig. 4b) but do not express oligodendrocyte markers such as Bcas1 or Olig1 (Fig. 4b). (PDF 255 kb) [file 12915_2017_383_MOESM7_ESM.pdf]
